# Item selection, scaling and construct validation of the Patient-Reported Inventory of Self-Management of Chronic Conditions (PRISM-CC) measurement tool in adults

**DOI:** 10.1007/s11136-022-03165-4

**Published:** 2022-06-27

**Authors:** George Kephart, Tanya Packer, Åsa Audulv, Yu-Ting Chen, Alysia Robinson, Ingrid Olsson, Grace Warner

**Affiliations:** 1 Department of Community Health and Epidemiology, Dalhousie University, 5790 University Avenue, Halifax, NS B3H 1V7 Canada; 2 School of Health Administration, Dalhousie University, Halifax, Canada; 3 School of Occupational Therapy, Dalhousie University, Halifax, Canada; 4 Department of Nursing, Umeå University, Umeå, Sweden

**Keywords:** Patient outcome assessment, Self-management, Multimorbidity, Psychometrics, Chronic disease, Patient-centered care

## Abstract

**Purpose:**

To select and scale items for the seven domains of the Patient-Reported Inventory of Self-Management of Chronic Conditions (PRISM-CC) and assess its construct validity.

**Methods:**

Using an online survey, data on 100 potential items, and other variables for assessing construct validity, were collected from 1055 adults with one or more chronic health conditions. Based on a validated conceptual model, confirmatory factor analysis (CFA) and item response models (IRT) were used to select and scale potential items and assess the internal consistency and structural validity of the PRISM-CC. To further assess construct validity, hypothesis testing of known relationships was conducted using structural equation models.

**Results:**

Of 100 potential items, 36 (4–8 per domain) were selected, providing excellent fit to our hypothesized correlated factors model and demonstrating internal consistency and structural validity of the PRISM-CC. Hypothesized associations between PRISM-CC domains and other measures and variables were confirmed, providing further evidence of construct validity.

**Conclusion:**

The PRISM-CC overcomes limitations of assessment tools currently available to measure patient self-management of chronic health conditions. This study provides strong evidence for the internal consistency and construct validity of the PRISM-CC as an instrument to assess patient-reported difficulty in self-managing different aspects of daily life with one or more chronic conditions. Further research is needed to assess its measurement equivalence across patient attributes, ability to measure clinically important change, and utility to inform self-management support.

**Supplementary Information:**

The online version contains supplementary material available at 10.1007/s11136-022-03165-4.

## Plain English summary

Most of the daily work to manage life with chronic health conditions such as diabetes, heart disease, mental illness, arthritis, and many others is done by patients and their families. We know that this kind of self-management improves peoples’ health outcomes and quality of life. Assessment tools are needed to help people and health providers identify the kind of difficulties people are experiencing so that appropriate programs, resources and supports can be accessed. Unfortunately, existing assessment tools do not measure areas of difficulty most important to people and are inadequate for people who are living with multiple conditions. This study presents a new assessment tool to fill this gap and evaluates its validity. The Patient-Reported Inventory of Self-Management of Chronic Conditions (PRISM-CC) assesses patients’ perceived difficulty in self-managing seven different aspects of life with chronic health conditions. This study provides evidence that PRISM-CC measures these domains. The PRISM-CC is now ready for testing in clinical settings.

## Introduction

Enabling people to better self-manage their chronic conditions is widely regarded as critical to improving patient outcomes and alleviating the growing demand for medical care [1, 2]. Self-management is defined by the Institute of Medicine, as the daily tasks that “individuals must undertake to live well with one or more chronic conditions” [3]. Self-management is more than just medical management such as monitoring symptoms and adhering to therapies. It also includes management of emotions, roles, social relationships and daily activities [4–9]. Self-management of the different tasks is reciprocal; management of broader areas impacts ability to effectively manage symptoms, and vice-versa [5, 8, 10]. Self-management strategies and needed support change over time as chronic conditions and life circumstances change [1, 11–14].

Feasible, valid, and reliable measures that pinpoint patients’ self-management difficulties are required to guide patient care, and research on effectiveness of self-management interventions. Two scoping reviews of 35 existing self-management measures have identified major deficiencies in existing measures [15, 16]. First, the majority were developed for specific health conditions [15, 16]. While these have a role, generic measures relevant to persons living with multiple chronic conditions are needed to inform integrated, interdisciplinary, and client-centered care [5, 14, 17]. Second, most self-management measures do not differentiate aspects of self-management shown to be important to patients, nor the self-management supports required [15, 16, 18]. Most measures are unidimensional, providing only a single score across domains or limiting focus to specific aspects of self-management [16, 18]. Among the multidimensional measures, all lack content validity when assessed against reviews of self-management tasks important to patients [15]. Finally, many existing measures also lack a cohesive theoretical or conceptual framework [15, 16].

The Patient-Reported Inventory of Self-Management of Chronic Conditions (PRISM-CC) is a new, patient-reported instrument designed to address the limitations of existing measures [19]. Designed to be applicable to patients living with multiple chronic conditions, it will differentiate domains of self-management relevant to patient experiences, and be feasible for use in diverse settings. Its intended purpose is to inform individualized self-management support plans, program evaluation, and research on chronic disease management.

The conceptual foundation of the PRISM-CC is a validated, patient-centered framework describing seven domains of self-management strategies relevant to the experience of patients with one or more chronic conditions: the Taxonomy of Every Day Self-Management Strategies (TEDSS) [9, 10]. The TEDSS was first developed using concept mapping of research studies, then further refined and validated using longitudinal, qualitative data from 117 interviews with individuals with neurological conditions [7]. Given the diversity of age of onset, varied symptoms and unpredictable trajectory of neurological conditions, these data were well suited to refining and validating the TEDSS framework. It was also compared and found to be consistent with other conceptual frameworks developed for patients with other chronic conditions [1, 5, 20, 21].

The PRISM-CC is a patient-reported measure of perceived success (or difficulty) in self-managing each of the seven TEDSS domains (Table 1): Resource, Process, Internal, Activity, Social Interactions, Healthy Behaviors, and Disease Controlling domains. This paper reports item selection, scaling, and construct validation of the PRISM-CC. Our objectives were to: (1) select and scale items to measure the seven domains of the PRISM-CC, and (2) assess the internal consistency and construct validity of the PRISM-CC.

**Table 1:** Domain definitions, selected items and descriptive statistics (*N* = 1055)

Domain and item		Response categories (difficulty — Success)^a^							
		0	1	2	3	4	5	N/A	Missing
		*N* (%)	*N* (%)	*N* (%)	*N* (%)	*N* (%)	*N* (%)	*N* (%)	*N* (%)
Resource	*Self-perceived success in seeking, pursuing and/or managing needed formal or informal supports and resources*								
Res1	When I have appointments with my healthcare providers, I tell them what I want or need	19 (1.80)	42 (3.98)	101 (9.57)	271 (25.69)	381 (36.11)	223 (21.14)	16 (1.52)	2 (0.19)
Res2	I talk to my healthcare provider(s) about my condition(s)	23 (2.18)	60 (5.69)	93 (8.82)	224 (23.13)	384 (36.40)	244 (23.13)	10 (0.95)	17 (1.61)
Res3	I arrange appointments with my health care provider(s)	13 (1.23)	45 (4.27)	85 (8.06)	243 (23.03)	378 (35.83)	264 (25.02)	15 (1.42)	12 (1.14)
Res4	When I need to, I find people to help me understand information I receive about my condition(s)	34 (3.22)	57 (5.40)	104 (9.86)	243 (23.03)	350 (33.18)	158 (14.98)	96 (9.10)	13 (1.23)
Process	*Self-perceived success in seeking information, being aware of choices and making good decisions*								
Pro1	I identify what information I can trust	9 (0.85)	22 (2.09)	65 (6.16)	241 (22.84)	479 (45.40)	228 (21.61)	9 (0.85)	2 (0.19)
Pro2	I make informed decisions	2 (0.19)	31 (2.94)	82 (7.77)	241 (22.84)	444 (42.09)	249 (23.60)	3 (0.28)	3 (0.28)
Pro3	I think about the consequences of different decisions	6 (0.57)	28 (2.65)	56 (5.31)	263 (24.93)	462 (43.79)	229 (21.71)	9 (0.85)	2 (0.19)
Pro4	I try different things to find out what works best for me	9 (0.85)	24 (2.27)	94 (8.91)	344 (32.61)	410 (38.86)	147 (13.93)	25 (2.37)	2 (0.19)
Pro5	I keep myself updated with new information related to my health conditions	13 (1.23)	35 (3.32)	76 (7.20)	239 (22.65)	416 (39.43)	212 (20.09)	53 (5.02)	11 (1.04)
Internal	*Self-perceived success in creating inner calm by preventing and managing stress, negative emotions, and internal distress*								
Int1	I set realistic expectations for myself	36 (3.41)	107 (10.14)	166 (15.73)	393 (37.25)	268 (25.40)	69 (6.64)	11 (1.04)	5 (0.47)
Int2	I accept the things I cannot change	45 (4.27)	127 (12.04)	196 (18.58)	404 (38.29)	201 (19.05)	78 (7.39)	1 (0.09)	3 (0.28)
Int3	I manage my emotions and reactions	28 (2.65)	94 (8.91)	180 (17.06)	432 (40.95)	233 (22.09)	81 (7.68)	3 (0.28)	4 (0.38)
Int4	I have and use ways to recover after a bad day	28 (2.65)	60 (5.69)	169 (16.02)	431 (40.85)	282 (26.73)	61 (5.78)	17 (1.61)	7 (0.66)
Int5	I deal with frustration caused by my health situation	20 (1.90)	116 (11.00)	194 (18.39)	442 (41.90)	189 (17.91)	63 (5.97)	27 (2.56)	4 (0.38)
Int6	I manage my stress	45 (4.27)	105 (9.95)	227 (21.52)	434 (41.14)	188 (17.82)	50 (4.74)	2 (0.19)	4 (0.38)
Int7	I focus on the positives	27 (2.56)	91 (8.63)	145 (13.74)	341 (32.32)	323 (30.62)	120 (11.37)	3 (0.28)	5 (0.47)
Int8	I forgive myself when I make a mistake	36 (3.41)	131 (12.42)	218 (20.66)	370 (35.07)	211 (20.00)	82 (7.77)	0 (0.00)	7 (0.66)
Activity	*Self-perceived success in participating in everyday activities (leisure activities, work activities, household chores)*								
Act1	I organize things in my home to make my life easier	15 (1.42)	59 (5.59)	127 (12.04)	273 (25.88)	369 (34.98)	155 (14.69)	53 (5.02)	4 (0.38)
Act2	I plan ahead before going somewhere to be sure I can manage my health condition(s)	18 (1.71)	34 (3.22)	104 (9.86)	318 (30.14)	356 (33.74)	155 (14.69)	65 (6.16)	5 (0.47)
Act3	I plan my time so I can get things done	18 (1.71)	65 (6.16)	149 (14.12)	350 (33.18)	334 (31.66)	119 (11.28)	16 (1.52)	4 (0.38)
Act4	I manage my health condition(s) so that I can do things I enjoy	29 (2.75)	71 (6.73)	178 (16.87)	417 (39.53)	231 (21.90)	112 (10.62)	12 (1.14)	5 (0.47)
Act5	I make time to do things I enjoy	28 (2.65)	41 (3.89)	136 (12.89)	345 (32.70)	340 (32.23)	153 (14.50)	7 (0.66)	5 (0.47)
Social Interaction	*Self-perceived success in disclosing health issues, managing social interactions and relationships*								
Soc1	I prioritize social interactions that I enjoy	39 (3.70)	56 (5.31)	99 (9.38)	265 (25.12)	398 (37.73)	178 (16.87)	20 (1.90)	0 (0.00)
Soc2	I can explain my symptoms so family and friends can understand them	40 (3.79)	75 (7.11)	130 (12.32)	306 (29.00)	295 (27.96)	181 (17.16)	21 (1.99)	7 (0.66)
Soc3	I clearly express my needs to others	34 (3.22)	107 (10.14)	226 (21.42)	359 (34.03)	225 (21.33)	85 (8.06)	10 (0.95)	9 (0.85)
Soc4	I devote time and attention to those who are dear to me	14 (1.33)	42 (3.98)	83 (7.87)	235 (22.27)	390 (36.97)	271 (25.69)	13 (1.23)	7 (0.66)
Soc5	When problems with my health arise, I stay in touch with people who are important to me	32 (3.03)	88 (8.34)	134 (12.70)	300 (28.44)	303 (28.72)	163 (15.45)	28 (2.65)	7 (0.66)
Healthy Behavior	*Self-perceived success maintaining a healthy lifestyle in order to enhance health and limit the risk of lifestyle related illness*								
Hea1	I maintain healthy lifestyle behaviours that I know are important for my health	41 (3.89)	78 (7.39)	171 (16.21)	421 (39.91)	245 (23.22)	92 (8.72)	4 (0.38)	3 (0.28)
Hea2	I make healthy food choices	23 (2.18)	75 (7.11)	150 (14.22)	370 (35.07)	312 (29.57)	113 (10.71)	6 (0.57)	6 (0.57)
Hea3	I find ways to train my brain to keep mentally fit	31 (2.94)	51 (4.83)	106 (10.05)	289 (27.39)	372 (35.26)	185 (17.54)	18 (1.71)	3 (0.28)
Hea4	I create healthy sleeping habits	72 (6.82)	115 (10.90)	200 (18.96)	339 (32.13)	228 (21.61)	93 (8.82)	6 (0.57)	2 (0.19)
Hea5	I create time in my day to be active (walk to work, do housework, yard work or other daily activities)	45 (4.27)	85 (8.06)	174 (16.49)	368 (34.88)	246 (23.32)	123 (11.66)	10 (0.95)	4 (0.38)
Disease Controling	*Self-perceived success in managing health conditions including managing medications and treatments, monitoring symptoms and limiting complications*								
Dis1	When problems with my health arise, I understand what to do to manage my condition(s)	28 (2.65)	28 (2.65)	85 (8.06)	284 (26.92)	383 (36.30)	236 (22.37)	3 (0.28)	8 (0.76)
Dis2	I know what to do if I experience side-effects or other problems with my treatment or medication	16 (1.52)	53 (5.02)	71 (6.73)	204 (19.34)	376 (35.64)	258 (24.45)	70 (6.64)	7 (0.66)
Dis3	I know which symptoms I need to act upon	14 (1.33)	33 (3.13)	59 (5.59)	263 (24.93)	407 (38.58)	256 (24.27)	16 (1.52)	7 (0.66)
Dis4	I know what to do when my symptoms get worse	17 (1.61)	65 (6.16)	104 (9.86)	286 (27.11)	369 (34.98)	203 (19.24)	4 (0.38)	7 (0.66)

*NA* not applicable

^a^All items used the difficulty–easy response scale, except for the 4 Disease items which used the Strongly disagree–strongly agree response scale

## Methods

### Preliminary item generation

Development of the PRISM-CC followed the Patient-Reported Outcomes Measurement Information System (PROMIS)^®^ Scientific Standard [19] and the COSMIN Study Design checklist for Patient-Reported outcome measures [22–24]. The first step in development was to operationally reframe the seven TEDSS domain definitions to reflect perceived success or difficulty in self-managing each domain (Table 1). Item generation (Phase 1) and assessment of relevance and understanding to people living with chronic conditions (Phase 2) are previously reported [19]. Briefly, item development and selection were conducted by an international, multidisciplinary study team, which included clinicians, researchers and people living with chronic conditions. Potential items (*N* = 250) were generated for each domain using qualitative data employed to develop the TEDSS, and by examining items from other tools [9, 10]. After an initial conceptual review, 231 items were retained and administered to 40 persons living with chronic conditions to assess their relevance and understandability. Poorly performing items were discarded. Moderately performing items were further assessed during face-to-face cognitive interviews with ten of the 40 participants [25, 26]. We retained 100 items (11–17 items per domain—none from existing scales) for item selection, scaling, and validation.

### Participants and data collection

Between February 2020 and April 2021 1,213 persons aged 18 years and over, who self-reported having at least one chronic condition, and who could read English, completed an anonymous, 20–30-min online survey. To be eligible, subjects had to select at least one of 13 conditions from a list (see footnote, Table 2) or specify “other” conditions in a free-text field. Recruitment sought a diverse sample with respect to sociodemographic characteristics and types of chronic conditions through posters displayed in health care settings; information distributed to patient groups, health charities and organizations; social media (Facebook and Twitter), and online advertising sites (e.g., Kijiji). Some (*N* = 159) were also recruited using Amazon Mechanical Turk (https://www.mturk.com). We excluded 158 persons (13%) who had missing data on 50% or more of the items, for a final sample size of 1055. Those excluded were not statistically different than those included with respect to age, gender, household type, number of reported chronic conditions, self-reported health status, or self-reported mental health; however, they had lower educational attainment.

**Table 2:** Demographic and clinical characteristics of study participants (*N* = 1055)

Characteristic	*N* (%)
Age categories	
18–30 years	354 (33.55)
31–40 years	152 (14.41)
41–50 years	87 (8.25)
51–60 years	89 (8.44)
61+ years	268 (25.40)
Missing	105 (9.95)
Gender identity	
Female	719 (68.15)
Male	300 (28.44)
Other	35 (3.32)
Missing	1 (0.09)
Country of residence	
Canada	582 (55.17)
USA	245 (23.22)
Other	223 (21.14)
Missing	5 (0.47)
Living situation	
Live alone	260 (24.64)
Shared household	744 (70.52)
Shelter/homeless/facility/other	46 (4.36)
Missing	5 (0.47)
Highest level of education completed	
Less than high school	30 (2.84)
High school	201 (19.11)
Post-secondary degree	558 (52.89)
Graduate degree	257 (24.36)
Missing	9 (0.85)
Self-reported chronic conditions^a^	
Arthritis	231 (10.72)
Bowel disorder	219 (10.17)
Cancer	34 (1.58)
Cardiovascular disease	138 (6.41)
Diabetes	179 (8.31)
Lung disease	108 (5.01)
Neurological disorder	137 (6.36)
Psychiatric disorder	678 (31.48)
Other	430 (19.96)
Count of chronic conditions	
1	442 (41.90)
2	298 (28.25)
3+	315 (29.86)

^a^Collapsed from self-reported list with response options: arthritis, Alzheimer’s or dementia, bipolar disorder, bowel disorder, cancer, depression, diabetes, effects of stroke, heart disease, lung disease, neurological disorder (e.g., multiple sclerosis), schizophrenia, other psychiatric disorders, other (please specify)

The study procedure adhered to the Canadian Tri-Council Policy Statement on Ethical Conduct for Research Involving Humans, and ethics approval was obtained from the Nova Scotia Health Research Ethics Board (Romeo File #: 1025263). Informed consent was obtained from all survey respondents.

### Measures

The survey included 100 potential PRISM-CC items in two versions with different item orders. The preferential response scale assessed difficulty: unable to do, with extreme difficulty, with much difficulty, with a little difficulty, easily, very easily (66 items). When this was not appropriate, two other response scales were used: (1) never, rarely, sometimes, often, almost always, always (24 items), and (2) strongly disagree, disagree, moderately disagree, moderately agree, agree, strongly agree (10 items). For each item, high scores indicated self-management success. Each item included a “not applicable” (NA) response option.

The survey also included sociodemographic questions (age, gender, country of residence, current living situation, and educational status), type(s) of chronic conditions, self-reported general health and mental health status, a question on impact of chronic condition(s) on daily life, and the 6-item Self-Efficacy in Managing Chronic Conditions (SEMCD) measure [27]. The SEMCD is widely used to evaluate patients’ confidence in managing chronic conditions and is strongly correlated with other measures of self-management [18, 28]. Self-reported general (and mental health) were measured with a single question “In general, would you say your (mental) health is”: excellent, very good, good, fair, or poor [29–31]. Impact on daily life was measured with the question: “Overall, how much do you feel that your chronic condition(s) affect(s) your life?”: not at all, a little bit, moderately, quite a bit, or extremely.

### Analytic approach

As per our published protocol [19], analyses proceeded through item selection, model refinement and then assessment of construct validity. Confirmatory factor analysis (CFA) and item response theory (IRT) models provided complementary approaches to inform item selection, scaling, and assessment of construct validity of the PRISM-CC [32, 33]. Models appropriate for ordinal response scales were employed. CFA models were estimated using the R ‘Lavaan’ package based on pairwise polychoric correlations and diagonally weighted least squares [34, 35]. For IRT, multidimensional graded response models were estimated with full information maximum likelihood using the R ‘mirt’ package [36, 37].

Missing data resulted from both item non-response and “NA” responses. Items with high proportions of “NA” responses resulting from life contexts uncommon to many respondents (e.g., managing chronic conditions at work) were excluded; however, missing and “NA” responses are unlikely to be missing completely at random [38]. While our estimation methods handled item non-response, sensitivity analyses were conducted to assess the impact of non-random patterns of missingness on reported model parameters and fit statistics. Multivariate chained equations were used to impute 10 sets of missing and “NA” item responses using the “mice v2.9” package in R using all items in our final model as predictors [39]. Parameter estimates and fit statistics were then re-estimated and pooled across the 10 chained imputation data sets, and compared to our reported results.

The item selection process identified the best performing set of items to create a parsimonious measurement instrument for the seven PRISM-CC domains while differentiating between domains. Item selection proceeded through six iterations of modeling and item assessment. Only a few items were excluded at each iteration, and models were refitted before further decisions were made. At later iterations, items previously discarded were reconsidered by adding them back into more refined model versions to evaluate how they performed. Item selection decisions considered multiple statistical criteria combined with ongoing assessment of face and content validity by our interdisciplinary team. Cognitive interview data and qualitative analyses, which informed item development, were often used to inform decisions. Statistical information informing item selection decisions included: (1) low item variance or very low item-rest correlations; (2) high rates of item non-response or not applicable responses resulting from context-specific items; (3) weak standardized factor loadings (< 0.6) or weak discrimination parameters in the IRT models (< 1.35) [40, 41]; (4) examination of IRT item location parameters, item-category response function plots and item information plots to assess the contribution of each item to measuring the latent variable; (5) indications of problems with item performance based on the 1–2 largest modification indices at each model iteration, such as correlated errors (which violates the conditional independence assumption) or cross-loading of an item between domains (indicative of weak discriminant validity of an item); (6) evidence of differential item functioning by sociodemographic variables; and (7) areas of local strain based on residual correlations. A more detailed description of how each of these criteria were used is provided in Supplementary Appendix.

Once items for the PRISM-CC were selected, we assessed construct validity by assessing the extent to which data corresponded to our conceptual framework (structural validity), and hypothesis testing based on known relationships [42].

Structural validity was assessed by the fit of the data to correlated domain CFA and IRT measurement models reflecting our full conceptual framework, and individual measurement models for each of the seven domains. Internal consistency of each domain was assessed with Cronbach’s alpha and measures of marginal reliability. To assess global fit of models, we computed the Root Mean Square Error of Approximation (RMSEA), Squared Root Mean Residual (SRMR), Tucker Lewis Index (TLI) and Comparative Fit Index (CFI). Standard cut-offs widely used in the literature as indicative of good fit were used (SRMR < 0.05, RMSEA < 0.06, and TLI and CFI > 0.95) [43]. However, a RMSEA of 0.06 can indicate poor fit in a model with low standardized factor loadings (e.g., 0.40) while a RMSEA of 0.20 can indicate good fit in a model with high standardized factor loadings (e.g., > 0.90) [40]. So, we also assessed the fit of individual items using CFA standardized factor loadings, IRT item discrimination parameters, and the infit and outfit mean square fit statistics. Standardized factor loadings in the range 0.60–0.74 were interpreted as high, and values ≥ 0.75 were interpreted as very high [40]. IRT item discrimination parameters in the range 1.35–1.69 were considered high, and those ≥ 1.70 were considered very high [41]. Good infit and outfit statistics have values close to 1.0 and should be in the range of 0.5 to 1.5 [44]. Residuals between observed and model predicted inter-item correlations were examined to identify local areas of poor fit. To further assess structural validity, discrimination between latent variables was assessed by testing for collapsibility of domains that were highly correlated (≥ 0.80).

Non-reflective responses in online surveys can result in underestimation of model fit [45, 46]. Of the 1,055 respondents included in our analysis, 5% spent, on average, less than 3.6 s per question, giving plausibility to this concern. Accordingly, sensitivity analyses were conducted to assess the potential impact of non-reflective responses on the model fit of each PRISM-CC domain (see Supplementary Appendix).

Finally, as detailed in our protocol [19], we further assessed construct validity of the PRISM-CC using structural equation models to test hypothesized associations between each domain and variables previously shown to be associated with self-management. Briefly, we hypothesized that the SEMCD would be positively associated with all domains; higher education would be associated with higher Process, Resource, and Healthy Behaviors domain scores; a higher number of chronic conditions would be associated with lower Disease Controlling domain scores; higher self-perceived general health would be associated with higher Disease Controlling domain scores; and higher self-perceived mental health would be associated with higher Internal and Social Interaction domain scores.

## Results

The sample included respondents with a diverse range of chronic conditions and social circumstances (Table 2). Younger adults and females are likely overrepresented relative to the adult populations of persons with chronic conditions, and a large proportion of respondents, especially those who were younger reported mental health conditions. Chronic conditions such as cardiovascular disease and chronic obstructive pulmonary disease (COPD) are likely underrepresented relative to adult populations of persons with chronic conditions, while many rarer chronic conditions are likely overrepresented.

### Item selection

Thirty-six items, with 4–8 items per domain, were selected for the PRISM-CC (Table 1). Early in the selection process, items were most frequently eliminated because of poor item-rest correlations and weak factor loadings, combined with evidence of poor face validity. Later in the item selection process, content validity (e.g., redundant items), poor discriminant validity, and evidence of differential item functioning where more common reasons for item elimination. We selected items for each domain that best captured the construct while resulting in very good to excellent model fit. See Supplemental Appendix for detailed description of the item selection.

Face and content validity of selected items was assessed by our team to be high for all domains except the Resource domain, which was assessed as moderate because some content of the domain definition was not captured. None of the selected domain items explicitly ask about locating or using community and/or social services, or informal supports, though the fourth item may do so implicitly. Two potential items about self-perceived difficulty in finding “community” and “social” services were eliminated due to ambiguity in meaning. Potential items to measure difficulty accessing informal supports (e.g., “When I need to, I ask family or friends to help me.”) were eliminated due to poor discriminant validity with the Social Interaction domain.

### Construct validity and internal consistency

Evidence for the structural validity of the PRISM-CC is strong. The data provided excellent fit to our hypothesized correlated factors CFA model and corresponding multidimensional IRT graded response model. The seven factor CFA model had an RMSEA of 0.057 (90% CI 0.055–0.060), an SRMR of 0.039, a CFI of 0.951, and a TLI of 0.947. Table 3 shows that items for all domains had high measurement quality. All standardized loadings were high or very high (19 of 36 are very high), and nearly all IRT graded response item discrimination parameters were very high (34 of 36). Infit and outfit statistics were generally close to 1.0, and all met fit criteria. Measurement quality for items in the Healthy Behaviors domain was weaker, on average, than other domains.

**Table 3:** Item parameters and fit statistics for the correlated factor model and multidimensional graded response model

Domain and item	Correlated factor model			Graded response model				
	loading	SE	Std. loading	Discrim	Std. loading	Item diff	Outfit	Infit
Resource								
Res1	1.000		0.812	2.553	0.832	− 1.13	0.922	0.951
Res2	1.002	0.026	0.813	2.869	0.860	− 1.00	0.803	0.895
Res3	0.914	0.026	0.742	2.087	0.775	− 1.25	0.980	0.928
Res4	1.038	0.028	0.842	2.344	0.809	− 0.94	0.973	0.983
Process								
Pro1	1.000		0.658	1.877	0.741	− 1.63	0.917	0.993
Pro2	1.250	0.041	0.822	3.034	0.872	− 1.37	0.933	1.013
Pro3	1.039	0.039	0.683	1.927	0.749	− 1.66	0.952	1.029
Pro4	1.168	0.045	0.768	1.922	0.749	− 1.53	1.088	1.078
Pro5	1.136	0.041	0.747	1.880	0.741	− 1.52	1.050	1.068
Internal								
Int1	1.000		0.809	2.210	0.792	− 0.54	0.979	0.977
Int2	0.901	0.022	0.729	2.166	0.786	− 0.35	0.920	0.934
Int3	1.005	0.020	0.813	2.720	0.848	− 0.54	0.908	0.927
Int4	0.994	0.021	0.804	2.335	0.808	− 0.72	0.988	1.006
Int5	0.983	0.021	0.795	2.303	0.804	− 0.45	0.998	1.013
Int6	0.988	0.020	0.799	2.654	0.842	− 0.34	0.953	0.954
Int7	0.989	0.020	0.800	2.391	0.815	− 0.71	0.927	0.927
Int8	0.923	0.021	0.746	2.159	0.785	− 0.33	0.899	0.907
Activity								
Act1	1.000		0.715	1.867	0.739	− 1.07	0.983	1.011
Act2	1.003	0.030	0.718	1.896	0.744	− 1.19	0.922	0.956
Act3	1.084	0.032	0.775	2.394	0.815	− 0.82	0.924	0.941
Act4	1.168	0.035	0.835	2.669	0.843	− 0.66	0.992	0.993
Act5	1.113	0.034	0.796	2.426	0.819	− 0.95	0.981	1.005
Social Interaction								
Soc1	1.000		0.708	1.781	0.723	− 1.05	1.007	1.010
Soc2	1.048	0.033	0.742	2.003	0.762	− 0.81	0.912	0.942
Soc3	1.072	0.031	0.759	2.029	0.766	− 0.45	0.951	0.954
Soc4	0.980	0.033	0.694	1.806	0.728	− 1.36	0.950	0.969
Soc5	1.084	0.035	0.767	2.298	0.804	− 0.74	0.881	0.911
Healthy Behavior								
Hea1	1.000		0.807	2.698	0.846	− 0.59	0.892	0.941
Hea2	0.827	0.024	0.667	1.704	0.707	− 0.79	0.882	0.902
Hea3	0.882	0.026	0.712	1.537	0.670	− 1.29	1.012	1.062
Hea4	0.821	0.026	0.663	1.537	0.670	− 0.37	0.994	1.009
Hea5	0.892	0.023	0.720	1.885	0.742	− 0.64	0.973	0.991
Disease Controlling								
Dis1	1.000		0.820	2.354	0.810	− 1.24	1.039	1.037
Dis2	0.807	0.035	0.662	1.904	0.746	− 1.26	0.904	0.964
Dis3	0.903	0.039	0.740	2.347	0.810	− 1.37	0.909	0.981
Dis4	0.991	0.035	0.812	2.485	0.825	− 1.02	0.991	0.982

Evidence of model fit and internal consistency was also assessed individually for each PRISM-CC domain (Table 4). Internal consistency, as measured by Cronbach’s alpha and marginal reliability, exceeded 0.80 for all domains. The CFI, TLI and SRMR fit statistics indicated excellent fit for all domains in both CFA and IRT models. However, the upper confidence intervals for RMSEAs for both the CFA and IRT models were higher than the commonly used cut-off of 0.06. However, examination of residual correlations did not indicate localized areas of poor fit in any domain, and modification indices did not identify substantial model misspecifications. Sensitivity analyses (see Supplemental Appendix) indicated that non-reflective responses resulted in underestimation of the fit of the PRISM-CC. Dropping potentially non-reflective respondents increased standardized factor loadings by 0.05 or more, and substantially improved indices of model fit, including the RMSEA. Sensitivity analyses re-estimating all CFA models using multiply imputated missing and not applicable responses resulted in small changes to standardized factor loadings and fit statistics but did not alter any study conclusions.

**Table 4:** Estimated Cronbach’s alpha, fit indices and marginal reliability by PRISM-CC domain

	Resource	Process	Internal	Activity	Social Interaction	Healthy Behavior	Disease Controlling
Cronbach's alpha	0.848	0.819	0.914	0.854	0.829	0.801	0.809
Confirmatory factor analysis models							
CFI	1.000	0.995	0.995	0.992	0.985	0.995	0.994
TLI	0.999	0.991	0.993	0.984	0.999	0.989	0.988
RMSEA	0.025	0.063	0.057	0.091	0.065	0.065	0.065
90% LCI	0.000	0.044	0.045	0.069	0.042	0.042	0.043
90% UCI	0.071	0.088	0.069	0.116	0.090	0.090	0.090
SRMR	0.007	0.019	0.023	0.020	0.020	0.020	0.022
Graded response models							
Reliability	0.859	0.843	0.924	0.869	0.846	0.859	0.831
C2 RMSEA	0.000	0.026	0.045	0.071	0.053	0.057	0.000
95% LCI	0.000	0.000	0.032	0.049	0.030	0.034	0.000
95% UCI	0.040	0.055	0.058	0.096	0.078	0.082	0.051
SRMR	0.013	0.025	0.026	0.029	0.025	0.032	0.039

*CFI* Comparative Fit Index, *TLI* Tucker–Lewis index, *RMSEA* root mean square error of approximation, *SRMR* standardized root mean squared residuals, *LCI* lower confidence interval, *UCI* upper confidence interval

Estimated correlations between PRISM-CC domains were positive and exceeded 0.50, for both the CFA and IRT graded response models (Table 5). The Healthy Behaviors domain had particularly high estimated correlations with the Internal (~ 0.85) and the Activity (~ 0.87) domains. However, the conceptual distinction between these domains was clear, and statistical tests for each pair, comparing a two-factor model with a constrained one-factor model, indicated better fit for the two-factor models. Furthermore, modification indices for the CFA model did not identify unspecified cross loadings contributing to high correlations between these domains.

**Table 5:** Estimated correlations between PRISM-CC domains and the between PRISM domains and the Self-Efficacy in Managing Chronic Conditions Scale (SEMCD)

	Resource	Process	Internal	Activity	Social Interaction	Healthy Behavior	Disease Controlling
Resource		0.781	0.628	0.700	0.793	0.605	0.615
Process			0.687	0.698	0.758	0.724	0.590
Internal				0.762	0.759	0.853	0.510
Activity					0.794	0.875	0.594
Social Interaction						0.741	0.545
Healthy Behavior							0.524
SEMCD	0.510	0.494	0.692	0.689	0.592	0.687	0.527

All reported correlations are significantly different than zero (*p* < 0.0001) and have narrow confidence intervals. Correlations between PRISM-CC domains are from a seven factor CFA model. Correlations between domains estimated from a multidimensional IRT graded response model (not shown) were nearly identical. Correlations between PRISM-CC domains and the SEMCD are from two-factor CFA models

Testing of pre-specified hypotheses provided further evidence of the construct validity. All hypotheses were confirmed (Tables 5 and 6). There were strong and statistically significant associations between the SEMCD and each of the PRISM-CC domains (Table 5). As shown in Table 6, higher education was associated with higher Process, Resource, and Healthy Behavior domain scores; a larger number of chronic conditions was associated with lower Disease Controlling domain scores; self-perceived general health was associated with higher Disease Controlling domain scores; and higher self-perceived mental health was associated with higher Internal and Social Interaction domain scores.

**Table 6:** Testing of hypothesized associations between PRISM-CC domains and other variables to assess construct validity

Predictor	Domain	Regression coefficient	Std. Error	95% CI
Education (ref: < post-secondary)	Resource	*p* value < 0.001		
Post-secondary degree		0.63	0.20	0.23, 1.03
Graduate degree		1.11	0.24	0.64, 1.58
Education (ref: < post-secondary)	Process	*p* value < 0.001		
Post-secondary degree		0.64	0.18	0.28, 0.99
Graduate degree		1.16	0.22	0.74, 1.58
Mental health (ref: poor)	Internal	*p* value < 0.001		
Fair		1.75	0.22	1.32, 2.18
Good		3.04	0.24	2.56, 3.51
Very good		4.12	0.28	3.58, 4.67
Excellent		5.24	0.34	4.56, 5.91
Mental health (ref: poor)	Social Interaction	*p* value < 0.001		
Fair		1.05	0.21	0.63, 1.46
Good		1.60	0.22	1.18, 2.03
Very good		2.38	0.24	1.91, 2.85
Excellent		3.37	0.31	2.77, 3.98
Education (ref: < post-secondary)	Healthy Behavior	*p* value < 0.001		
Post-secondary degree		1.30	0.32	0.67, 1.94
Graduate degree		2.05	0.38	1.29, 2.80
No. of reported conditions	Disease Controlling	*p* value < 0.001		
Continuous (1–7)		− 0.21	0.07	− 0.34, − 0.08
General health (ref: Poor)	Disease Controlling	*p* value < 0.001		
Fair		0.43	0.32	− 0.19, 1.05
Good		1.01	0.30	0.41, 1.60
Very good		1.52	0.33	0.87, 2.16
Excellent		2.37	0.45	1.48, 3.26
Participation restriction (ref: < moderately)	Activity	*p* value < 0.001		
Moderately		− 1.37	0.21	− 1.77, − 0.96
Quite a bit		− 2.19	0.22	− 2.62, − 1.76
Extremely		− 3.18	0.26	− 3.69, − 2.67

*p* values are based on the Wald chi-square test

*Std. Error* Standard error, *95% CI* 95% confidence interval of coefficient estimate, *ref* reference group

## Discussion

### Key Findings and Limitations

The PRISM-CC includes 36 items with 4–8 items per domain. All domains, with the partial exception of the Resource domain, have good face and content validity when measured against our conceptual framework. The operational definition of the Resource domain was “self-perceived success in seeking, pursuing and/or managing needed formal or informal supports and resources”. Items retained in this domain measure success in managing formal health care but do not explicitly capture success managing informal supports (help from family and friends) or accessing community and social services. Items generated to measure management of informal supports had poor discriminant validity with the Social Interaction domain, so were not retained. This suggests that management of informal supports, while important to patients, is indistinguishable from the management of social contacts and networks, which is captured by the Social Interaction domain. As well, items expected to measure difficulty managing community and social service supports performed poorly and were not retained due to possible differences among respondents in defining social and community services across different contexts.

Evidence of the PRISM-CC’s structural validity, one aspect of construct validity, is strong. Our data had very good to excellent fit to the pre-specified, correlated factor CFA and IRT models. While estimated upper confidence intervals for the RMSEA in many of the CFA and IRT domain-specific models were above the widely used cut-off of 0.06, we believe this is not of much concern. An RMSEA greater than 0.06 may indicate good fit when standardized factor loadings are greater than 0.70, which is the case for most of our items [40]. Moreover, sensitivity analyses of the potential impact of non-reflective responses to survey questions suggest that our estimates of standardized factor loadings and model fit are likely conservative relative to what would be obtained from application of the PRISM-CC in clinical settings.

Sample characteristics may limit the generalizability of results. While we had a large and diverse sample, data came from people living in the community, not from clinical settings. The online nature of the survey contributed to the younger cohort whose chronic disease patterns likely differ from older and/or clinical populations. To address this, further assessment of measurement equivalence across patients with different socio-demographic and chronic disease attributes is needed.

Confirmation of a priori, hypothesized associations between PRISM-CC domains and variables, as detailed in the study protocol [19], provides additional evidence of construct validity. While this is encouraging, additional evidence of validity is needed. The critical next step is to assess the ability of the PRISM-CC to inform patient care, measure clinically meaningful change, and evaluate the critical ingredients of self-management interventions.

While strong evidence for internal consistency of each domain was confirmed, assessment of test–retest reliability, measurement error and responsiveness of the PRISM-CC remains unassessed.

### Clinical utility

Self-management is the work done by people themselves [3, 5–7]. The TEDSS articulates that work in seven domains, identified and described by people living with chronic conditions and labelled using non-medical language [9, 10]. Built on this foundation, the PRISM-CC is expected to be understandable, intuitive, and relevant to patients’ experiences. It is not a measure of underlying ability or capacity, but rather patients’ personal assessment of their success/difficulty within each domain. For example, difficulty in the Disease Controlling domain represents perceived difficulty managing “medications and treatments, monitoring symptoms and limiting complications” regardless of whether it is the result of managing a complex medication and treatment regimen or having insufficient knowledge to manage a single medication. In either case, and regardless of the underlying reason, perceived difficulty is uncovered so that self-management support can be offered.

Measuring perceived difficulty, the PRISM-CC stands in contrast to tools that focus on single constructs, such as self-efficacy or patient activation, believed to bolster self-management [27, 47]. However, many other factors, such as health literacy, social support networks, access to transportation, use of technologies or tools, and the development of specific skills or strategies also enable self-management [48–50]. The PRISM-CC facilitates a patient-centered approach in which patients identify areas of difficulty. If indicated, contributing factors can then be investigated. Given that the success of most self-management support interventions is dependent on the actions of patients, and often necessitates behaviour change, patients are most likely to engage in interventions that are of perceived difficulty [51].

With its multi-dimensionality and focus on patient-perceived difficulty, the PRISM-CC is positioned to facilitate tailored interventions, addressing a concern with “one size fits all” self-management interventions [14, 52, 53]. Its seven domains explicate, from the perspective of the patient, strengths or challenges in self-managing traditional areas of medical (Disease Controlling and Healthy Behaviours domains), roles (Activities and Social Interaction domains) and emotional management (Internal domain) while also assessing problem solving (Process domain) and aspects of health navigation (Resource domain), providing valuable insight into how to tailor support [6]. For example, a newly diagnosed patient who identifies difficulty finding information, problem solving and decision-making (Process domain) but no difficulty with routine exercise and healthy food choices (Healthy Behaviours domain) needs different support than a patient with multiple conditions and limited mobility who self-reports greatest difficulty in the Activities and Social Interaction domains. Using the PRISM-CC to pinpoint areas of patient concern coupled with skillful clinical assessment, tailored interventions can be offered, and appropriate referrals made. Success or difficulty in some domains will likely cluster together, as indicated by the high correlations between them.

PRISM-CC also has the potential to improve the functioning and patient-centeredness of multidisciplinary and interprofessional teams. Assigning disciplinary case managers based on patient needs and provider scope of practice are two examples. This work could be strengthened using the Team Assessment of Self-Management Strategies (TASMS), also based on the TEDSS framework, to better understand the types of self-management support teams provide [54].

Finally, the PRISM-CC is expected to be feasible for both research and clinical settings, and for individuals living with one or multiple chronic conditions. It is short to administer (36 items) and does not need specific training. Visualized reporting tools are under development, and licensing and apps for administration and linkage to electronic clinical records are being investigated.

## Conclusions

The PRISM-CC was developed through a process grounded in a validated conceptual model, item generation and item selection. This study provides evidence for the internal consistency and construct validity of the PRISM-CC. Data from a large and diverse sample of adults with chronic conditions was found to have very good to excellent fit to the conceptual model. PRISM-CC domains were confirmed to have hypothesized associations with other measures and variables. The PRISM-CC holds promise for use in clinical care, program evaluation, and self-management research, but further validation in these settings is needed.

## Supplementary Information

Below is the link to the electronic supplementary material.

Supplementary file1 (PDF 167 kb)

## References

[1] Schulman-Green D, Jaser S, Martin F, Alonzo A, Grey M, McCorkle R, Redeker NS, Reynolds N, Whittemore R (2012). Processes of self-management in chronic illness. Journal of Nursing Scholarship.

[2] Van de Velde D, De Zutter F, Satink T, Costa U, Janquart S, De SD, Vriendt P (2019). Delineating the concept of self-management in chronic conditions: A concept analysis. British Medical Journal Open.

[3] Corrigan JM, Adams K, Greiner AC (2004). 1st Annual Crossing the Quality Chasm Summit: A Focus on Communities.

[4] Simmons LA, Wolever RQ, Bechard EM, Snyderman R (2014). Patient engagement as a risk factor in personalized health care: A systematic review of the literature on chronic disease. Genome Med.

[5] Liddy C, Blazkho V, Mill K (2014). Challenges of self-management when living with multiple chronic conditions: Systematic review of the qualitative literature. Canadian Family Physician.

[6] Corbin J, Strauss A (1985). Managing chronic illness at home: Three lines of work. Qualitative Sociology.

[7] Audulv Å, Packer T, Hutchinson S, Roger KS, Kephart G (2016). Coping, adapting or self-managing—what is the difference? A concept review based on the neurological literature. Journal of Advanced Nursing.

[8] Audulv A, Asplund K, Norbergh K-G (2012). The integration of chronic illness self-management. Qualitative Health Research.

[9] Audulv Å, Ghahari S, Kephart G, Warner G, Packer TL (2019). The Taxonomy of Everyday Self-management Strategies (TEDSS): A framework derived from the literature and refined using empirical data. Patient Education and Counselling.

[10] Audulv Å, Hutchinson S, Warner G, Kephart G, Versnel J, Packer TL (2021). Managing everyday life: Self-management strategies people use to live well with neurological conditions. Patient Education and Counselling.

[11] Grey M, Knafl K, McCorkle R (2006). A framework for the study of self- and family management of chronic conditions. Nursing Outlook.

[12] Audulv Å (2013). The over time development of chronic illness self-management patterns: A longitudinal qualitative study. BMC Public Health.

[13] Satink T, Cup EHC, de Swart BJM, Nijhuis-van der Sanden MWG (2015). How is self-management perceived by community living people after a stroke? A focus group study. Disability and Rehabilitation.

[14] Bratzke LC, Muehrer RJ, Kehl KA, Lee KS, Ward EC, Kwekkeboom KL (2014). Self-management priority setting and decision-making in adults with multimorbidity: A narrative review of literature. International Journal of Nursing Studies.

[15] Packer TL, Fracini A, Audulv Å, Alizadeh N, van Gaal BGI, Warner G, Kephart G (2018). What we know about the purpose, theoretical foundation, scope and dimensionality of existing self-management measurement tools: A scoping review. Patient Education and Counselling.

[16] Hudon É, Hudon C, Lambert M, Bisson M, Chouinard MC (2021). Generic self-reported questionnaires measuring self-management: A scoping review. Clinical Nursing Research.

[17] Hopman P, Schellevis FG, Rijken M (2016). Health-related needs of people with multiple chronic diseases: Differences and underlying factors. Quality of Life Research.

[18] Kephart G, Packer TL, Audulv Å, Warner G (2019). The structural and convergent validity of three commonly used measures of self-management in persons with neurological conditions. Quality of Life Research.

[19] Packer T, Kephart G, Audulv Å, Keddy A, Warner G, Peacock K, Sampalli T (2020). Protocol for development, calibration and validation of the Patient-Reported Inventory of Self-Management of Chronic Conditions (PRISM-CC). British Medical Journal Open.

[20] Schulman-Green D, Jaser SS, Park C, Whittemore R (2016). A metasynthesis of factors affecting self-management of chronic illness. Journal of Advanced Nursing.

[21] Eton DT, Ridgeway JL, Egginton JS, Tiedje K, Linzer M, Boehm DH, Poplau S, Ramalho de Oliveira D, Odell L, Montori VM, May CR, Anderson RT (2015). Finalizing a measurement framework for the burden of treatment in complex patients with chronic conditions. Patient Related Outcome Measures.

[22] PROMIS Instrument Development and Validation Scientific Standards Version 2.0 (revised May 2013).

[23] Gagnier JJ, Lai J, Mokkink LB, Terwee CB (2021). COSMIN reporting guideline for studies on measurement properties of patient-reported outcome measures. Quality of Life Research.

[24] Mokkink LB, de Vet HCW, Prinsen CAC, Patrick DL, Alonso J, Bouter LM, Terwee CB (2018). COSMIN risk of bias checklist for systematic reviews of patient-reported outcome measures. Quality of Life Research.

[25] Drennan J (2003). Cognitive interviewing: Verbal data in the design and pretesting of questionnaires. Journal of Advanced Nursing.

[26] Peterson CH, Peterson NA, Powell KG (2017). Cognitive interviewing for item development: Validity evidence based on content and response processes. Measurement and Evaluation in Counseling and Development.

[27] Lorig K, Chastain RL, Ung E, Shoor S, Holman HR (1989). Development and evaluation of a scale to measure perceived self-efficacy in people with arthritis. Arthritis and Rheumatism.

[28] Ritter PL, Lorig K (2014). The English and Spanish Self-Efficacy to Manage Chronic Disease Scale measures were validated using multiple using multiple studies. Journal of Clinical Epidemiology.

[29] Layes A, Asada Y, Kepart G (2012). Whiners and deniers—what does self-rated health measure?. Social Science and Medicine.

[30] Ahmad F, Jhajj AK, Stewart DE, Burghardt M, Bierman AS (2014). Single item measures of self-rated mental health: A scoping review. BMC Health Services Research.

[31] Bombak AE (2013). Self-rated health and public health: A critical perspective. Frontiers in Public Health.

[32] Brown TA (2015). Confirmatory factor analysis for applied research.

[33] Kamata A, Bauer DJ (2008). A note on the relation between factor analytic and item response theory models. Structural Equation Modeling: A Multidisciplinary Journal.

[34] Rosseel Y (2012). Lavaan: An R package for structural equation modeling and more. Version 0.5–12 (BETA). Journal of Statistical Software.

[35] Gana K, Broc G (2019). Structural equation modeling with Lavaan.

[36] Chalmers RP (2012). mirt: A multidimensional item response theory package for the R environment. Journal of Statistical Software.

[37] Reckase MD (2011). Multidimensional item response theory.

[38] Holman R, Glas CA, Lindeboom R, Zwinderman AH, de Haan RJ (2004). Practical methods for dealing with ‘not applicable’ item responses in the AMC Linear Disability Score project. Health and Quality of Life Outcomes.

[39] Buuren SV, Groothuis-Oudshoorn K (2011). Multivariate imputation by chained. Journal of Statistical Software.

[40] McNeish D, An J, Hancock GR (2018). The thorny relation between measurement quality and fit index cutoffs in latent variable models. Journal of Personality Assessment.

[41] Baker FB, Kim S-H (2017). The basics of item response theory using R.

[42] Mokkink LB, Terwee CB, Patrick DL, Alonso J, Stratford PW, Knol DL, Bouter LM, de Vet HC (2010). The COSMIN study reached international consensus on taxonomy, terminology, and definitions of measurement properties for health-related patient-reported outcomes. Journal of Clinical Epidemiology.

[43] Hu L, Bentler PM (1999). Cutoff criteria for fit indexes in covariance structure analysis: Conventional criteria versus new alternatives. Structural Equation Modeling: A Multidisciplinary Journal.

[44] Smith RM, Schumacker RE, Bush MJ (1998). Using item mean squares to evaluate fit to the Rasch model. Journal of Outcome Measurement.

[45] Beck MF, Albano AD, Smith WM (2019). Person-fit as an index of inattentive responding: A comparison of methods using Polytomous survey data. Applied Psychological Measurement.

[46] Schneider S, May M, Stone AA (2018). Careless responding in internet-based quality of life assessments. Quality of Life Research.

[47] Hibbard JH, Mahoney ER, Stockard J, Tusler M (2005). Development and testing of a short form of the patient activation measure. Health Services Research.

[48] Glasgow RE, Huebschmann AG, Krist AH, Degruy FV (2019). An Adaptive, Contextual, Technology-Aided Support (ACTS) system for chronic illness self-management. Milbank Quarterly.

[49] Hyman I, Shakya Y, Jembere N, Gucciardi E, Vissandjée B (2017). Provider- and patient-related determinants of diabetes self-management among recent immigrants: Implications for systemic change. Canadian Family Physician.

[50] Koetsenruijter J, van Eikelenboom N, van Lieshout J, Vassilev I, Lionis C, Todorova E, Portillo MC, Foss C, Serrano GM, Roukova P, Angelaki A, Mujika A, Knutsen IR, Rogers A, Wensing M (2016). Social support and self-management capabilities in diabetes patients: An international observational study. Patient Education and Counselling.

[51] Michie S, van Stralen MM, West R (2011). The behaviour change wheel: A new method for characterising and designing behaviour change interventions. Implementation Science.

[52] Coulter A, Entwistle VA, Eccles A, Ryan S, Shepperd S, Perera R (2015). Personalised care planning for adults with chronic or long-term health conditions. Cochrane Database System Reviews.

[53] Kastner M, Hayden L, Wong G, Lai Y, Makarski J, Treister V, Chan J, Lee JH, Ivers NM, Holroyd-Leduc J, Straus SE (2019). Underlying mechanisms of complex interventions addressing the care of older adults with multimorbidity: A realist review. British Medical Journal Open.

[54] Keddy AC, Packer TL, Audulv Å, Sutherland L, Sampalli T, Edwards L, Kephart G (2021). The Team Assessment of Self-Management Support (TASMS): A new approach to uncovering how teams support people with chronic conditions. Healthcare Management Forum.

