# Supplementary material for: Item selection, scaling and construct validation of the Patient-Reported Inventory of Self-Management of Chronic Conditions (PRISM-CC) measurement tool in adults
Source: Qual Life Res. 2022 Jun 27;31(9):2867–80. doi: 10.1007/s11136-022-03165-4 (PMC9356954; doi:10.1007/s11136-022-03165-4)
Supplement: Supplementary file 1 — Supplementary file1 (PDF 167 kb) [file 11136_2022_3165_MOESM1_ESM.pdf]

Supplementary Appendix

Detailed Description of Item Selection Process and Criteria

Because potential items were selected based on a strong theoretical framework and underwent extensive qualitative and conceptual assessment prior to being used in this study, even the first iteration of the models provided evidence of fit to the conceptual model, and only a few items performed poorly. For example, the first run of models with all items yielded Cronbach's alphas for the 7 domains ranging from .86 - .93, and SRMRs ranging from .054 - .093. Accordingly, the item selection process focused on model refinement to identify the best performing set of items providing a parsimonious measurement instrument capturing the full range of each PRISM-CC domain, and differentiating between domains. Item selection and model refinement proceeded through six iterations. No more than a few items were excluded at each iteration, and then models were refitted before further decisions were made. At later iterations, items discarded at earlier iterations were reconsidered by adding them back into more refined version of the models to see how they performed.

Decisions to remove items were based on consideration of multiple statistical criteria and reassessment of face and content validity by our interdisciplinary team. Cognitive interview data and qualitative analyses, which informed item development, were often used to guide decisions. Statistical information informing item selection decisions included:

1. Low item variance: In the first iteration we excluded four items that had low variance (including due to ceiling or floor effects), or that were very weakly correlated with other items in the domain.
2. High rates of item non-response or not applicable responses: We excluded items where a high rate of missing or "not applicable" responses likely resulted from circumstances not salient to many respondents. For example, a potential resource domain item "*When I need to, I access supports and resources to help deal with my health condition(s) at work* " pertains to work environments and thus is not applicable to many persons not in the workforce; and the potential activity domain item "*I use tools/aids/equipment to make everyday activities easier*" was not applicable to many respondents who did not perceive that they used tools/aids/equipment. We excluded five items in the resource domain and one item in the activity domain based on this criterion.
3. Weak standardized factor loadings (<0.6) or weak discrimination parameters in the

IRT models (<1.35): Supported by other conceptual and statistical criteria (especially # 4 below), this criterion contributed to about a third of the item exclusions.

4. IRT item information and response: The performance of each item was assessed through examination of threshold parameters, item category response function plots (plots of the estimated probability of choosing each response to each item by level of theta), and item information function plots. Specifically, we considered:
   a. Whether thresholds and response curves for each item showed that the latent variable (i.e. theta) was associated with probability of selecting each sequential ordinal response category, and that each response category discriminated between levels of the latent variable. All items that met criteria #1-#3 also satisfied this criterion.
   b. The extent to which thresholds, response curves and information for each item showed that the ordinal response categories measured a broad spectrum of the latent variable. All items satisfied this criterion. The difficulty response scale, which was used for most items, was particularly strong on this criterion.
   c. The extent to which the items selected in each domain had thresholds, response curves and precision to measure the full continuum of each latent variable. This criterion was most often used to choose between similar items. Because the primary clinical utility of the PRISM-CC is to identify patients with perceived difficulty in each domain, we were particularly attentive to choosing items contributing precision at the difficulty end of each latent variable.
5. Large modification indices (MIs): At each iterative stage, we considered the 1-2 largest modification indices to identify potential areas of model misspecification that indicated problems with item performance. MIs were thus used to guide item selection, and not to modify model specification (i.e., at no point did we add correlated errors or cross loading to the models to improve fit). While MIs are conditional on the model estimated, their use was appropriate given that even first iteration of the model provided evidence of fit to the conceptual model, and evidence from MIs were always considered along with other statistical and conceptual criteria. Specifically, we considered:
   a. MIs identifying that adding correlated errors between items in the same domain would improve fit. Correlated errors may indicate item redundancy, item methods effects, or items measuring a common trait other than the

domain. Correlated errors would also violate the local independence assumption of IRT. This criterion contributed to approximately a third of item exclusions.

b. MIs identifying that the addition of a cross-loading of an item to another domain would improve model fit. This was considered indicative of poor discriminant validity of an item, and resulted when item wording related to more than one domain. This criterion contributed to approximately 10% of item exclusions.

6. Evidence of differential item functioning (DIF) by sociodemographic variables: At later iterations, analysis of differential item functioning was used to inform item selection decisions. Specifically, we used CFA models to test for differences in factor loadings and thresholds by age group (18-30, 31-60, 61+), gender identification (male, female, other), and education (high school diploma or less, post-secondary trade or bachelor's degree, and graduate degree). Only one item was eliminated based on DIF (the social domain item "*I make good choices about the time I spend with others*" showed differential item discrimination by gender).
7. Areas of local strain based on residual correlations: At the last iteration of item selection and model refinement, we examined residual correlations between items to identify areas of weaker model fit, and then reviewed other statistical and qualitative evidence to explore why. No substantial areas of local strain were identified, and this criterion did not result in additional item selection decisions.
8. Translatability: As a Swedish version of the PRISM-CC is being developed and tested, translatability of PRISM-CC items (assessed through forwards and backwards translation) was considered in item selection (primarily when choosing between semantically similar items).

Supplemental Table Showing Item Response Location Parameters

Table S1

Discrimination and Item Response Location (Difficulty) Parameters for the Multidimensional Graded Response Model

		Location (difficulty) parameters				
Domain	Discrim.	b1	b2	b3	b4	b5
Resource						
Res1	2.553	-2.595	-1.952	-1.262	-0.302	0.891

Res2	2.869	-2.471	-1.737	-1.175	-0.372	0.773
Res3	2.087	-3.045	-2.111	-1.438	-0.453	0.775
Res4	2.344	-2.357	-1.714	-1.089	-0.175	1.137
Process						
Pro1	1.877	-3.422	-2.667	-1.843	-0.655	0.975
Pro2	3.034	-3.438	-2.214	-1.444	-0.512	0.733
Pro3	1.927	-3.646	-2.576	-1.862	-0.580	0.967
Pro4	1.922	-3.372	-2.573	-1.595	-0.204	1.358
Pro5	1.880	-3.196	-2.369	-1.609	-0.490	1.014
Internal						
Int1	2.210	-2.423	-1.443	-0.722	0.509	1.874
Int2	2.166	-2.316	-1.301	-0.524	0.743	1.818
Int3	2.720	-2.406	-1.451	-0.681	0.562	1.642
Int4	2.335	-2.500	-1.739	-0.878	0.451	1.899
Int5	2.303	-2.726	-1.448	-0.618	0.783	1.865
Int6	2.654	-2.133	-1.316	-0.478	0.815	1.979
Int7	2.391	-2.540	-1.534	-0.856	0.180	1.414
Int8	2.159	-2.434	-1.308	-0.461	0.686	1.775
Activity						
Act1	1.867	-3.162	-2.044	-1.184	-0.158	1.305
Act2	1.896	-2.983	-2.261	-1.394	-0.132	1.280
Act3	2.394	-2.717	-1.763	-0.957	0.132	1.423
Act4	2.669	-2.359	-1.609	-0.795	0.444	1.428
Act5	2.426	-2.488	-1.901	-1.073	0.027	1.218
Social Interaction						
Soc1	1.781	-2.582	-1.903	-1.253	-0.223	1.257
Soc2	2.003	-2.465	-1.701	-1.000	0.052	1.157
Soc3	2.029	-2.556	-1.514	-0.561	0.596	1.792
Soc4	1.806	-3.241	-2.295	-1.550	-0.501	0.815
Soc5	2.298	-2.446	-1.553	-0.906	0.054	1.168
Healthy Behavior						
Hea1	2.698	-2.193	-1.474	-0.746	0.473	1.556
Hea2	1.704	-3.013	-1.917	-1.039	0.287	1.719
Hea3	1.537	-3.007	-2.177	-1.355	-0.157	1.322
Hea4	1.537	-2.329	-1.429	-0.540	0.672	1.987
Hea5	1.885	-2.408	-1.614	-0.803	0.411	1.552
Disease Controlling						
Dis1	2.354	-2.463	-2.045	-1.394	-0.343	0.865
Dis2	1.904	-2.939	-1.969	-1.411	-0.517	0.814
Dis3	2.347	-2.790	-2.103	-1.566	-0.480	0.789
Dis4	2.485	-2.656	-1.736	-1.146	-0.217	0.982

Note: Discrim. = Discrimination parameter.

Sensitivity Analyses for Potentially Careless Responses

Careless or non-reflective responses in online surveys can result in underestimation of model fit and data which is missing not at random (MNAR).(1, 2) Of the 1,055 respondents included in our analysis, 5% spent, on average, less than 3.6 seconds per question, giving plausibility to this concern. Accordingly, sensitivity analyses were conducted to assess the potential impact of careless responses on the model fit of each PRISM-CC domain.

We used a procedure proposed by Hong and Cheng.(3) Two person-fit statistics were estimated to measure the plausibility of each respondent's responses given the IRT graded response model.(4) The first, Gpoly, is based on the number of polytomous Guttman errors for each respondent.(5) Responders were classified as "non-reflective" if they were in the top 5% of Gpoly values. The second, lzpoly, is the standardized log-likelihood of the respondent's response vector, which is expected to be asymptotically normally distributed. Low values indicate poor person fit, and we classified non-reflective respondents as those with the 5% lowest values. The 5% cut-off is somewhat arbitrary and does not differentiate the magnitude of potential non-reflectiveness. Accordingly, we also treated lzpoly as a continuous indicator of non-reflectiveness, which was used to weight the contribution of each respondent.

For sensitivity analyses, we assessed the improvement in global models fit indices and standardized factor loadings for each of the seven PRISM-CC domains after (1) dropping subjects who were classified as being non-reflective responders, and (2) weighting the data for each respondent according to the inverse of their normalized person fit, based on the lzpoly person fit statistic. Substantial improvements in model fit would indicate high potential impact of careless responses.

As shown in Table 1, non-reflective responses result in underestimation of the fit of the PRISM-CC. Dropping potentially non-reflective respondents increased standardized factor loadings by 0.05 or more, and substantially improved indices of model fit, including the RMSEA.

Supplemental Table S2. Impact of Three Methods of Adjusting for Non-Reflective Responses on Model Fit and Factor Loadings

		Standardized loadings			
**Resource**					
	Item	Unadjusted	Lzpoly	Gpoly	Lz-weighted
	res1	0.783	0.858	0.864	0.865
	res2	0.891	0.935	0.922	0.918
	res3	0.764	0.817	0.788	0.840
	res4	0.749	0.805	0.808	0.827
	RMSEA				
	value	0.025	0.000	0.034	0.000
	Lower CI	0.000	0.000	0.000	0.000
	Upper CI	0.071	0.027	0.080	0.000
	SRMR	0.007	0.002	0.008	0.000
**Process**		Standardized loadings			
	Item	Unadjusted	Lzpoly	Gpoly	Lz-weighted
	pro1	0.746	0.801	0.775	0.806
	pro2	0.856	0.895	0.877	0.891
	pro3	0.726	0.773	0.775	0.800
	pro4	0.674	0.75	0.723	0.763
	pro5	0.667	0.74	0.743	0.769
	RMSEA				
	value	0.063	0.039	0.054	0.020
	lower CI	0.040	0.011	0.030	0.000
	upper CI	0.088	0.067	0.080	0.050
	SRMR	0.019	0.013	0.018	0.008
**Internal**		Standardized loadings			
	Item	Unadjusted	Lzpoly	Gpoly	Lz-weighted
	int1	0.762	0.800	0.799	0.801
	int2	0.776	0.833	0.828	0.841
	int3	0.831	0.876	0.869	0.869
	int4	0.771	0.812	0.810	0.811
	int5	0.763	0.827	0.820	0.846
	int6	0.815	0.866	0.858	0.868
	int7	0.792	0.829	0.834	0.835
	int8	0.776	0.809	0.806	0.816
	RMSEA				
	value	0.057	0.051	0.053	0.038
	lower CI	0.045	0.038	0.041	0.025
	upper CI	0.069	0.064	0.066	0.051
	SRMR	0.018	0.016	0.017	0.011
**Activity**		Standardized loadings			
	Item	Unadjusted	Lzpoly	Gpoly	Lz-weighted
	act1	0.718	0.774	0.776	0.784

	act2	0.730	0.773	0.767	0.795
	act3	0.792	0.847	0.834	0.859
	act4	0.828	0.857	0.840	0.859
	act5	0.775	0.814	0.818	0.850
	RMSEA				
	value	0.091	0.085	0.091	0.060
	lower CI	0.069	0.062	0.068	0.038
	upper CI	0.116	0.110	0.116	0.085
	SRMR	0.023	0.021	0.023	0.012
**Social Interaction**		Standardized loadings			
	Item	Unadjusted	Lzpoly	Gpoly	Lz-weighted
	soc1	0.676	0.746	0.763	0.785
	soc2	0.734	0.790	0.790	0.814
	soc3	0.749	0.780	0.776	0.798
	soc4	0.708	0.757	0.767	0.785
	soc5	0.800	0.818	0.817	0.838
	RMSEA				
	value	0.065	0.056	0.054	0.042
	lower CI	0.042	0.033	0.030	0.018
	upper CI	0.090	0.083	0.080	0.069
	SRMR	0.020	0.018	0.017	0.011
**Healthy Behavior**		Standardized loadings			
	Item	Unadjusted	Lzpoly	Gpoly	Lz-weighted
	hea1	0.897	0.935	0.913	0.915
	hea2	0.739	0.798	0.791	0.814
	hea3	0.574	0.622	0.682	0.678
	hea4	0.606	0.659	0.691	0.696
	hea5	0.674	0.737	0.750	0.768
	RMSEA				
	value	0.065	0.046	0.058	0.013
	lower CI	0.043	0.021	0.033	0.000
	upper CI	0.090	0.074	0.085	0.046
	SRMR	0.022	0.015	0.018	0.008
**Disease Controlling**		Standardized loadings			
	Item	Unadjusted	Lzpoly	Gpoly	Lz-weighted
	dis1	0.764	0.822	0.827	0.840
	dis2	0.720	0.764	0.763	0.786
	dis3	0.782	0.851	0.830	0.841
	dis4	0.788	0.858	0.845	0.873
	RMSEA				
	value	0.000	0.000	0.000	0.000
	lower CI	0.000	0.000	0.000	0.000
	upper CI	0.033	0.054	0.047	0.000

SRMR	0.002	0.004	0.004	0.001

References

1. Beck, M. F., A. D. Albano, and W. M. Smith. 2019. Person-Fit as an Index of Inattentive Responding: A Comparison of Methods Using Polytomous Survey Data. *Appl Psychol Meas* 43: 374-387.
2. Schneider, S., M. May, and A. A. Stone. 2018. Careless responding in internet-based quality of life assessments. *Qual Life Res* 27: 1077-1088.
3. Hong, M. R., and Y. Cheng. 2019. Robust maximum marginal likelihood (RMML) estimation for item response theory models. *Behav Res Methods* 51: 573-588.
4. Tendeiro, J. N., R. R. Meijer, and A. S. M. Niessen. 2016. PerFit: An R package for person-fit analysis in IRT. *Journal of Statistical Software* 74: 1-27.
5. Molenaar, I. W. 1991. A weighted Loevinger H-coefficient extending Mokken scaling to multicategory items. *Kwantitatieve Methoden* 12: 97-117.
